# Uterine “twisting sign”: A new potential ultrasonographic soft marker for deep endometriosis

**DOI:** 10.1002/ijgo.70274

**Published:** 2025-06-14

**Authors:** Fabio Barra, Simone Ferrero, Umberto Perrone, Giulio Evangelisti, Alessandra Pulliero, Alberto Izzotti, Umberto Leone Roberti Maggiore, Stefano Bogliolo

**Affiliations:** ^1^ Unit of Obstetrics and Gynecology Genoa Italy; ^2^ Department of Health Sciences (DISSAL) University of Genoa Genoa Italy; ^3^ Academic Unit of Obstetrics and Gynecology IRCCS Ospedale Policlinico San Martino Genoa Italy; ^4^ Department of Neurosciences, Rehabilitation, Ophthalmology, Genetics, Maternal and Child Health (DiNOGMI) University of Genoa Genoa Italy; ^5^ Unit of Obstetrics and Gynecology San Paolo Hospital—ASL2 Savona Italy; ^6^ Unit of Mutagenesis and Cancer Prevention IRCCS Ospedale Policlinico San Martino Genoa Italy; ^7^ Department of Experimental Medicine (DIMES) University of Genoa Genoa Italy; ^8^ Gynecologic Oncology Fondazione IRCCS Istituto Nazionale Tumori di Milano Milan Italy

**Keywords:** deep endometriosis, posterior sliding sign, rectosigmoid endometriosis, transvaginal ultrasonography, twisting sign

## Abstract

**Objective:**

The objective of the current study was to evaluate the “twisting sign,” defined as uterine fundus rotation observed on transvaginal sonography (TVS), as a potential soft marker for deep endometriosis (DE) and its association with specific DE localizations and indirect signs.

**Methods:**

A prospective observational study was conducted at an endometriosis referral center. We enrolled 158 reproductive‐aged women with pelvic pain requiring specialist evaluation. Exclusion criteria included prior endometriosis diagnosis or conditions affecting uterine positioning, such as large myomas, uterine malformations, or previous pelvic surgery. Standardized TVS assessments, following IDEA (International Deep Endometriosis Analysis) criteria, were performed by a single experienced operator. The twisting sign was defined as a uterine rotation angle between 15° and 90° in the fundal transverse section.

**Results:**

The twisting sign was detected in 24.1% of participants and was significantly associated with posterior compartment DE, particularly rectosigmoid nodules (44.8.7% vs. 14.0%, *P* < 0.001) and uterosacral ligament involvement (41.4% vs. 23.3%, *P* = 0.046). It was also linked to indirect DE markers, including ovarian fixation to the uterine wall (37.9% vs. 19.4%, *P* = 0.031) and absence of the posterior sliding sign (37.9% vs. 9.3%, *P* < 0.001). Multivariate analysis confirmed the twisting sign as an independent predictor of rectosigmoid junction nodules (odds ratio [OR], 9.84 [95% confidence interval [CI], 1.69–58.83], *P* = 0.012) and absence of the posterior sliding sign (OR, 6.63 [95% CI, 1.88–24.34], *P* = 0.004).

**Conclusion:**

The twisting sign may represent a novel and potentially valuable ultrasonographic marker of DE, particularly in the posterior pelvic compartment. It likely reflects mechanical distortion of the uterine axis due to DE nodules and adhesions. Multicenter validation is warranted.

## 1 INTRODUCTION

Pelvic endometriosis, defined by the presence of endometrial‐like tissue outside the uterine endometrium and myometrium, is a benign chronic disease affecting approximately 10% of reproductive‐aged women, with prevalence rising to 35%–50% among symptomatic individuals. It is categorized into three phenotypes: peritoneal, ovarian, and deep endometriosis (DE). DE, present in up to 20% of women with pelvic endometriosis, is a severe form characterized by the infiltration of fibrous and muscular tissues beneath the peritoneum. It commonly involves the uterosacral ligaments, rectosigmoid colon, vagina, bladder, and parametrium, causing significant morbidity.^1^

Transvaginal sonography (TVS) remains the cornerstone imaging technique for the evaluation and diagnosis of endometriosis.^2^ In 2016, the IDEA (International Deep Endometriosis Analysis) group developed a consensus to standardize the sonographic evaluation of DE, highlighting the role of “soft markers”—including site‐specific tenderness, ovarian immobility, and the posterior sliding sign—as indirect indicators of deep disease.^3^, ^4^ These markers, detectable via TVS, may raise suspicion of an underlying deep disease.^2^

Despite advances in the sonographic evaluation of DE, the orientation and positioning of the uterus—particularly in relation to posterior DE lesions—remain underexplored. In a normal uterus, the endometrial echo appears as a continuous line from cervix to fundus in the median longitudinal plane, regardless of uterine version. However, in endometriosis, fibrotic retraction and adhesions may alter the uterine axis, causing rotation or deviation from the expected transverse alignment.^5^ Such distortion is especially likely in the presence of posterior DE nodules, which can mechanically “pull” the uterine fundus into an abnormal position.

Given the well‐documented association between DE and pelvic anatomical distortion,^6^ this study aims to describe, for the first time, uterine axis rotation visible on TVS as a novel indirect marker of disease. To our knowledge, no previous studies have systematically evaluated fundal rotation on the transverse plane as a potential soft marker of DE. By addressing this gap, we propose the “twisting sign”—a reproducible finding on routine TVS—as another potential indirect indicator of posterior disease. Its identification may enhance diagnostic accuracy and confidence, particularly in preoperative planning for women with suspected endometriosis.

## 2 MATERIALS AND METHODS

This prospective study included women of reproductive age referred to the Chronic Pelvic Pain and Endometriosis Center, ASL‐4 Liguria (October 2022–July 2024), for pain requiring a specialist evaluation. The primary objective was to describe the prevalence and characteristics of the twisting sign, an abnormal uterine fundus rotation, as a potential ultrasonographic marker for DE. The secondary objective was to evaluate its correlation with specific DE localizations and indirect ultrasonographic markers. Patients were excluded if they had prior surgical, ultrasonographic, or radiological diagnoses of pelvic endometriosis. Other exclusion criteria included a history of pelvic inflammatory disease, prior retroperitoneal or uterine surgery (excluding diagnostic or operative hysteroscopy and uterine dilation and curettage), or the presence of medium to large myomas (>3 cm) or congenital uterine malformations, which could impact the assessment of uterine rotation.

Participants underwent a clinical assessment documenting demographics, medical treatments, and symptoms, including pain and gastrointestinal or urinary issues. Subsequently, patients underwent a standardized ultrasonographic evaluation performed by a single experienced operator (F.B.), who conducts more than 200 TVS annually.

### 2.1 Ultrasound

Two high‐performance ultrasonographic machines (Affinity 70, Philips, and HERA W9, Samsung) were equipped with a 5‐ to 9‐MHz transvaginal and a 3.5‐ to 5‐MHz transabdominal probes.

The evaluation of uterine fundus rotation was conducted based on a previously reported method.^7^

**FIGURE 1:**
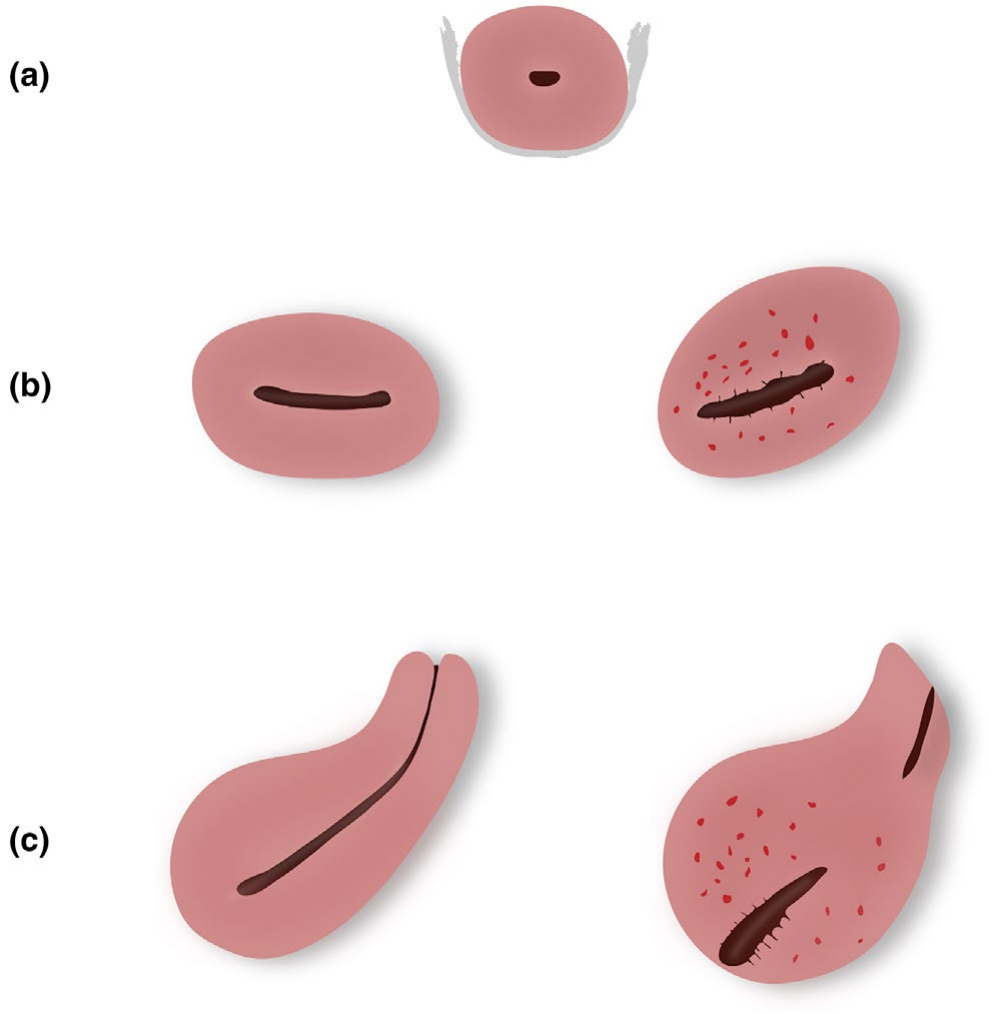
Illustration of the uterine “twisting sign” observed during transvaginal ultrasonography. (a) Transverse section of the cervical canal showing hyperechoic uterosacral ligaments in a semi‐horizontal orientation. (b) With caudal tilting of the probe, the upper uterine cavity is visualized. The left image illustrates a negative twisting sign, where the uterine rotation angle is negligible, and the endometrial echo appears horizontal relative to the plane. In contrast, the right image depicts a positive twisting sign, characterized by a uterine rotation angle of 15°–90° and an inclined endometrial echo, allowing quantification of the rotation. (c) Median longitudinal plane: The left image demonstrates a negative twisting sign, with an uninterrupted endometrial echo clearly visible. Conversely, the right image illustrates a positive twisting sign, where the endometrial echo appears interrupted, requiring probe rotation for continuous visualization.

**FIGURE 2:**
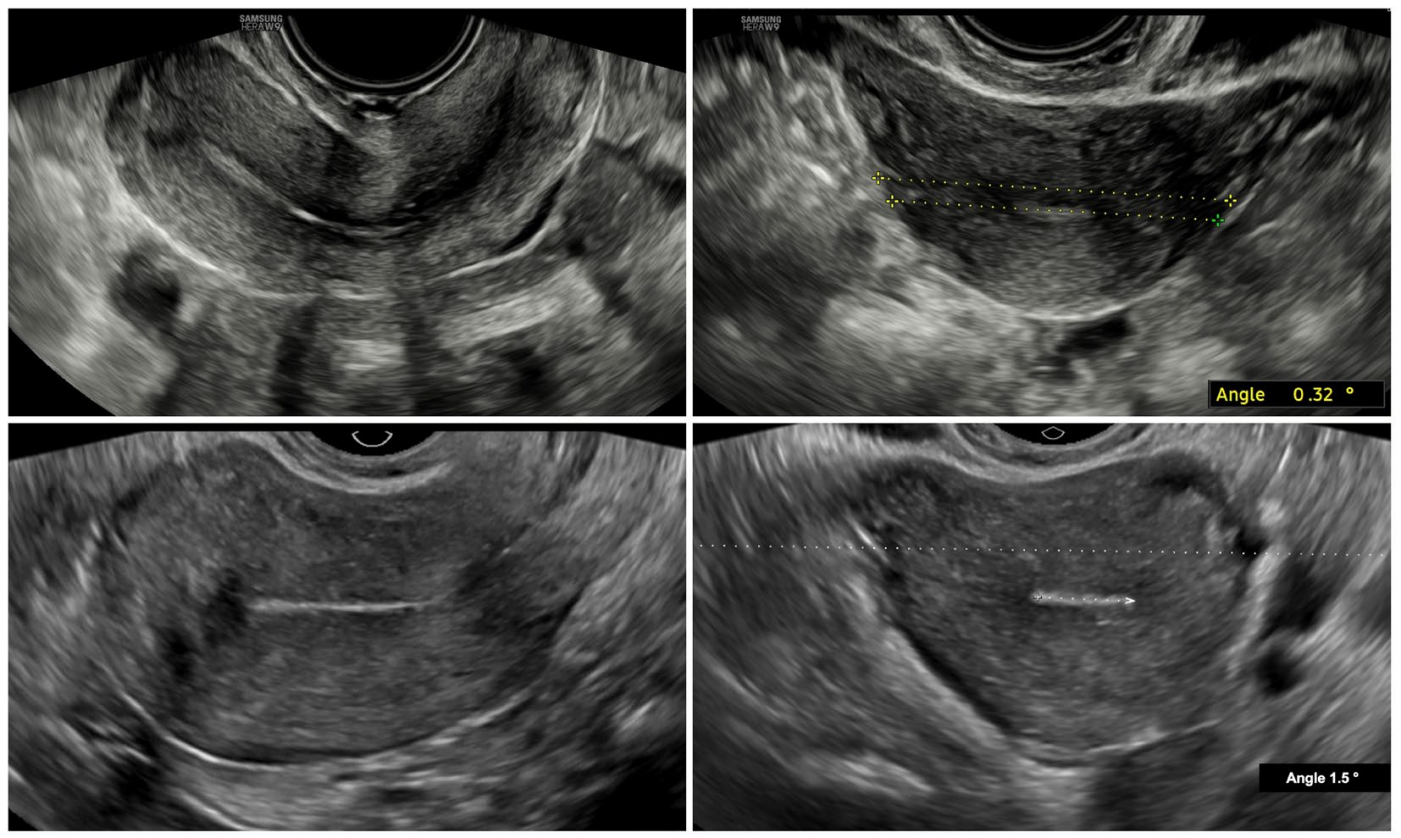
Physiological uteri (left: Longitudinal views; right: Transverse views) without evidence of pelvic endometriosis. The “twisting sign” is absent (uterine rotation angle <15°), indicating a lack of uterine axis rotation.

**FIGURE 3:**
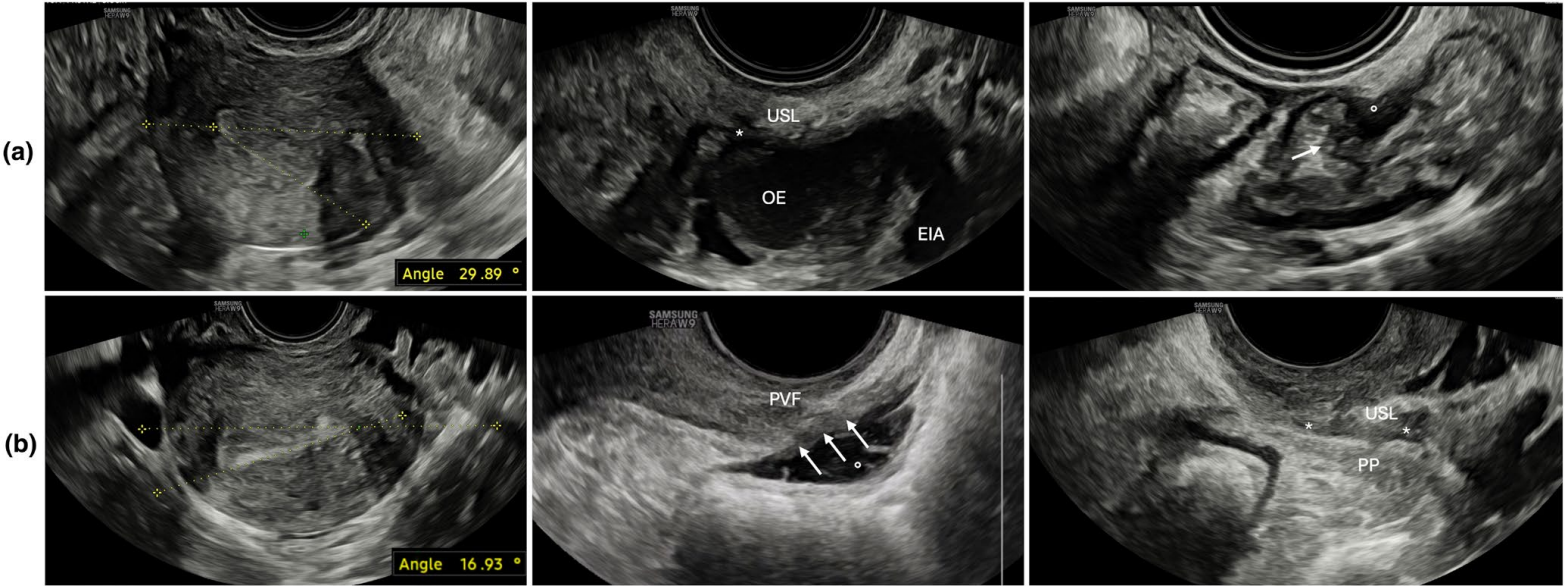
Presence of the “twisting sign” with uterine axis rotation to the left (29.9°; case a) and to the right (16.9°; case b). Case (a): Notable findings include a left ovarian endometrioma (OE) adherent to the left uterine wall and a deep endometriotic nodule (*) in the left uterosacral ligament (USL). In addition, an 8 × 6‐mm rectosigmoid junction nodule (°) is visible, causing visceral retraction and deep muscular infiltration, with initial invasion into the submucosal layer (arrow). Case (b): The image shows a large infiltrating nodule in the upper and lower rectum (22 × 11 mm;°) extending into the rectovaginal septum (arrows) and the posterior vaginal fornix (PVF). Another deep endometriotic nodule (*) is visible in the right USL, with further extension into the ipsilateral posterior parametrium (PP). The posterior sliding sign is absent, indicating extensive posterior compartment adhesions and obliteration of the Pouch of Douglas. In both cases, the uteri show direct and indirect signs of adenomyosis (hyperechogenic islands, myometrial cysts, and globular shape). The posterior sliding sign is absent, consistent with partial/complete obliteration of the posterior pelvic compartment. EIA, external iliac artery.

The transvaginal probe was inserted into the vagina up to the level of the cervix and then gently retracted slightly to minimize pressure on the cervix. Initially, a transverse section of the middle third of the cervical canal was obtained. In this view, the uterosacral ligaments appear as hyperechoic linear structures extending laterally from the cervix in a semi‐horizontal orientation.^8^ The probe was then cranially advanced to capture the upper third of the uterine cavity, maintaining the endometrial echo clearly visible. The angle between a transverse line drawn across the imaging plane and a tangential line along the endometrial echo was measured in proximity of the interostial line, referred to as the uterine rotation angle. The twisting sign was considered positive if this rotation angle fell between 15° and 90°. In addition, the direction of rotation, whether towards the right or left, was noted (Figures 1, 2, 3).

To further verify the presence of the twisting sign, the endometrial profile was assessed in a median longitudinal plane by maintaining the transvaginal probe with the reference point at the 12‐o'clock position. Unlike in a normally positioned uterus—where the endometrial line in the median longitudinal plane tends to be seen uninterrupted from the cervix to the fundus—a twisted uterus requires probe rotation to fully capture the endometrial sequence, as previously documented.^5^ This method allows for a rough but effective confirmation of the twisting sign (Videos S1 and S2).

The presence and characterization of pelvic endometriosis were evaluated according to the IDEA consensus statement.^9^ The ultrasonographic evaluation systematically identified indirect DE signs by adhering to the initial three steps (I, II, and III) of the consensus.

This process involved assessing the uterus and adnexa for ultrasonographic signs of uterine adenomyosis and the presence of endometriomas (IDEA step I). According to the revised MUSA (Morphological Uterus Sonographic Assessment) criteria,^10^ a diagnosis of adenomyosis was made if at least one of the direct features (myometrial cysts, hyperechogenic islands, or subendometrial lines or buds) was identified. Adenomyosis was classified as focal or diffuse depending on whether more or less than 25% of the lesion's circumference was surrounded by normal myometrium. The severity of adenomyosis was also classified based on the percentage of the overall affected myometrium (mild <25%, moderate 25%–50%, severe >50%).^11^

Endometriomas often present as unilocular cysts with ground glass echogenicity within the cyst fluid.^12^ The evaluation of soft markers of DE (IDEA step II) included assessing site‐specific ovarian tenderness and mobility. This was achieved by applying pressure between the ovary and the uterus; the ovary could appear hypomobile or fixed if it did not glide smoothly against the uterus, pelvic sidewall, or the contralateral ovary, as seen in the case of “kissing ovaries”.^13^ The status of the Pouch of Douglas (POD) and of the anterior vesical‐uterine plica was assessed through real‐time ultrasound–based analysis of the “sliding sign” (IDEA step III).

A thorough assessment of anatomical pelvic regions was conducted by evaluating the anterior (bladder) and posterior (rectosigmoid, uterosacral ligaments, rectovaginal septum, and vagina) pelvic compartments for the presence of DE nodules (IDEA step IV). Pertinent information, such as the number, size, and precise localization of DE nodules, was meticulously gathered. Cases of contiguous DE lesions extending across multiple pelvic structures were treated as distinct instances, as previously described in our research.^14^ On ultrasound, DE nodules typically appear as hypoechoic or heterogeneous lesions with indistinct margins, often infiltrating adjacent structures. They may exhibit hyperechoic foci, representing fibrosis or calcifications, and are frequently associated with retraction or adhesions. The specific appearance varies depending on their location.^9^

The evaluation of DE nodules in the anterior, lateral, and posterior parametrium was conducted following the characterization and topographic anatomy previously published by our research group,^8^ as confirmed by a recent IDEA addendum.^15^ Ureteric dilatation in the distal third of both ureters due to an extrinsic or intrinsic involvement by endometriosis^16^ was evaluated and supplemented with abdominal ultrasonography to assess hydronephrosis.

### 2.2 Ethical approval

The last study protocol was approved by the local ethics committee (N. Comitato Etico Territoriale Liguria: 295/2024—database identifier ID 13965). Patients participating in the study provided written informed consent. This study followed the STROBE (Strengthening the Reporting of Observational Studies in Epidemiology) checklist for cohort study (File S1).^17^

### 2.3 Statistical analysis

Data were analyzed with SPSS software version 26.0 (IBM). A *P* value <0.05 was considered statistically significant.

Descriptive statistics were calculated for all variables and appropriately expressed as continuous or categorical data. Continuous variables were compared using the Student *t* test or Mann–Whitney *U* test, while categorical variables were analyzed using the *χ* ^2^ test or Fisher exact test.

Multivariate logistic regression was performed to identify independent predictors of the twisting sign, including variables significant in univariate analysis (*P* < 0.05) or those deemed clinically relevant. Results are expressed as odds ratios (ORs) with 95% confidence intervals (CIs), adjusting for factors such as previous abdominal surgery, hormonal therapy, parity, and infertility.^18^ Pearson or Spearman correlation coefficients assessed the relationship between uterine rotation angle and various endometriotic localizations.

No formal a priori sample size calculation was performed because of the exploratory nature of the study. However, based on the observed frequency of the twisting sign and its association with posterior compartment DE, a post hoc power analysis was conducted to assess the adequacy of the sample size.

## 3 RESULTS

### 3.1 Demographic characteristics

The final analysis included 158 women (see Study Flowchart; Figure S1) with a mean age of 35.9 ± 9.0 years. Most participants were nulliparous (*n* = 126; 79.7%). Chronic pelvic pain was reported by 109 women (69.0%), dyspareunia by 97 (72.4%), and dysmenorrhea by 89 (81.7%). Gastrointestinal symptoms were present in 74 participants (46.8%). Hormonal treatment was administered to 104 women (65.8%), primarily in the form of combined oral contraceptives (*n* = 53; 51.0%). Surgical treatment was deemed necessary for 28 patients (17.7%). Table 1 provides a detailed summary of the demographic characteristics of the study population.

**TABLE 1:** Demographic characteristics of the study population.

	*N* = 158
Age (years)	35.9 ± 9.0
Body mass index (kg/m^2^)	25.2 ± 4.3
Race	
White	149 (94.3%)
Black	6 (3.8%)
Asian	3 (1.9%)
Smokers	15 (9.5%)
Previous parity	32 (20.3%)
Infertility	20 (12.7%)
Any previous abdominal surgery	36 (22.8%)
Symptoms	
Prevalence of dysmenorrhea	89/109 (81.7%)^a^
Intensity of dysmenorrhea	5.2 ± 2.3
Prevalence of deep dyspareunia	97/134 (72.4%)^b^
Intensity of deep dyspareunia	4.9 ± 2.1
Prevalence of chronic pelvic pain	109 (69.0%)
Intensity of nonmenstrual pelvic pain	4.7 ± 2.0
Prevalence of gastrointestinal symptoms	74 (46.8%)
Dyschezia	32 (20.3%)
Constipation	46 (29.1%)
Diarrhea	27 (17.1%)
Abdominal bloating	41 (25.9%)
Intestinal cramping	37 (23.4%)
Passage of mucus	23 (14,6%)
Hormonal therapy at time of study	104 (65.8%)
Estroprogestins	60 (57.7%)
Oral sequential use	35 (58.3%)
Oral continuous use	18 (30.0%)
Vaginal ring	6 (10.0%)
Transdermal patch	1 (1.7%)
Progestins	39 (37.5%)
Desogestrel	17 (43.6%)
Dienogest	2 (5.1%)
Drospirenon	7 (18.0%)
Norethindrone acetate	2 (5.1%)
Etonogestrel subdermal implant	5 (12.8%)
Levonorgestrel‐releasing intrauterine device	6 (15.4%)
GnRH analogs	3 (2.9%)
GnRH antagonists	2 (1.9%)

*Note*: Values are presented as number (percentage) or mean ± standard deviation. GnRH, gonadotropin‐releasing hormone.

^a^ All other patients were using hormonal therapies causing amenorrhea.

^b^ Forty‐three patients were not sexually active.

### 3.2 Ultrasonographic data

Ultrasound imaging confirmed evidence of DE nodules in 58 patients (36.7%). Specifically, rectosigmoid endometriosis was identified in 31 patients (19.6%). Uterine adenomyosis was described in 39 patients (24.7%); among these, diffuse features were described in 89.7% of cases (mild, 52.1%; moderate, 18.4%; severe, 17.1%). Ovarian endometriomas were detected in 26 patients (16.5%), but the kissing ovaries sign was seen in only four patients (2.5%). The absence of the posterior sliding sign was noted in 23 patients (14.5%). Infiltration of posterolateral parametrium was described in 3.8% (*n* = 6) of cases. Table 2 outlines the various localizations of endometriosis observed during ultrasonographic evaluation.

**TABLE 2:** Ultrasonographic findings associated with the presence of the “twisting sign”.

	Evidence of the twisting sign	No evidence of the twisting sign	Total	*P* value
Endometriomas (any side)	8 (27.6%)	18 (14.0%)	26 (16.5%)	0.074
Focal adenomyosis	0 (−)	4 (3.1%)	4 (2.5%)	0.337
Diffuse adenomyosis	10 (34.5%)	25 (19.4%)	35 (22.2%)	0.077
DE nodules				
Upper/lower rectum	5 (17.2%)	10 (7.8%)	15 (9.5%)	0.115
Rectosigmoid junction	6 (20.7%)	5 (3.9%)	11 (7.0%)	0.001
Sigmoid	2 (6.9%)	4 (3.1%)	6 (3.8%)	0.334
Uterosacral ligaments (any side)	12 (41.4%)	30 (23.3%)	42 (26.6%)	0.046
Rectovaginal septum	1 (3.4%)	2 (1.6%)	3 (1.9%)	0.499
Posterolateral parametrium (any side)	1 (3.4%)	5 (3.9%)	6 (3.8)	0.913
Vagina	1 (3.4%)	2 (1.6%)	3 (1.9%)	0.499
Bladder	1 (3.4%)	1 (0.8%)	2 (1.3%)	0.245
Indirect DE signs				
Ovarian fixity to pelvic wall (any side)	2 (6.9%)	11 (8.5%)	13 (8.2%)	0.773
Ovarian fixity to uterus (any side)	11 (37.9%)	25 (19.4%)	36 (22.8%)	0.031
Absence of anterior sliding sign	1 (3.4%)	6 (4.7%)	7 (4.4%)	0.776
Absence of posterior sliding sign	11 (37.9%)	12 (9.3%)	23 (14.6%)	<0.001
“Kissing ovaries”	0 (−)	4 (3.4%)	4 (2.5%)	0.337
Hydronephrosis (any side)	1 (3.4%)	1 (0.8%)	2 (1.3%)	0.245

*Note*: Values are presented as number (percentage). DE, deep endometriosis.

The twisting sign was identified in 38 patients (24.1%). Among these, uterine rotation was predominantly to the right (61.1%) compared with the left (38.9%), with a mean rotation angle of 20.3° ± 4.4°. There was no significant correlation between the presence of the twisting sign and the ultrasonographic measurements of the uterus (Table S1).

Patients who exhibited the twisting sign demonstrated a significantly higher prevalence of DE nodules in the posterior pelvic compartment (31.5% vs. 11.5%; *P* = 0.002). Based on the observed prevalences, the post hoc power analysis yielded a power of 76.6% (*α* = 0.05, two‐sided) to detect a statistically significant difference between groups, supporting the adequacy of the sample for exploratory analysis. In particular, 44.8% and 41.4% of patients with the twisting sign showed rectosigmoid and uterosacral ligament (any side) DE nodules, respectively, compared with 14.0% and 23.3% of those without the sign (*P* < 0.001 and *P* = 0.046). Similarly, diffuse uterine adenomyosis with moderate to severe extension was more frequently observed in the twisting sign group (24.1% vs. 7.8%; *P* = 0.010). Among the 42 patients (26.6%) who underwent surgical evaluation following TVS, the twisting sign was observed in 10 cases, of which nine (90.0%) had intraoperative confirmation of posterior compartment DE (rectosigmoid and/or uterosacral ligament with or without torus involvement). Conversely, among the 32 patients without the twisting sign, posterior DE was confirmed intraoperatively in only six cases (18.8%).

After adjustments for potential confounding factors, the multivariate logistic regression analysis confirmed that the twisting sign was an independent predictor of posterior compartment DE nodules (Table 3), particularly those involving the rectosigmoid junction (OR, 9.84 [95% CI, 1.69–58.83]; *P* = 0.012). In addition, patients with the twisting sign were more likely to exhibit indirect sonographic signs of endometriosis, such as a fixed ovary adhering to the uterine surface (37.9% vs. 19.4%; *P* = 0.031) and a negative posterior sliding sign (37.9% vs. 9.3%; *P* < 0.001). The absence of the posterior sliding sign was strongly predicted by the presence of the twisting sign (OR, 6.63 [95% CI, 1.88–24.34]; *P* = 0.004). There was a moderate significant positive correlation between the degree of uterine rotation and the absence of the posterior sliding sign (*r* = 0.328, *P* < 0.001) and a weak but significant positive correlation with the total number of posterior DE nodules (*r* = 0.231, *P* = 0.004). However, no significant correlation was found between the side of uterine rotation and the laterality of DE nodules (*r* = 0.121, *P* = 0.245).

**TABLE 3:** Concomitant ultrasonographic findings in patients with or without the “twisting sign” (multivariate analysis).

Ultrasonographic evidence	OR	95% CI
Endometriomas (any side)	1.92	0.51–6.78
Focal adenomyosis	0.72	0.15–5.62
Diffuse adenomyosis	1.55	0.51–4.60
DE nodules		
Upper/lower rectum	2.60	0.51–13.97
Rectosigmoid junction	9.84	1.69–58.83
Sigmoid	0.83	0.66–10.72
Uterosacral ligaments (any side)	1.27	0.46–3.93
Rectovaginal septum	1.79	0.12–48.11
Posterolateral parametrium (any side)	0.61	0.010–25.33
Vagina	0.52	0.014–48.80
Bladder	0.14	0.022–34.57
Indirect DE signs		
Ovarian fixity to pelvic wall (any side)	0.25	0.02–2.51
Ovarian fixity to uterine wall (any side)	2.13	0.72–7.26
Absence of anterior sliding sign	3.18	0.60–39.81
Absence of posterior sliding sign	6.63	1.88–24.34
“Kissing ovaries”	1.11	0.70–5.75
Hydronephrosis (any side)	0.94	0.0045–200.81

Abbreviations: CI, confidence interval; DE, deep endometriosis; OR, odds ratio.

## 4 DISCUSSION

The current study demonstrates that the twisting sign, a novel potential ultrasonographic marker, is significantly associated with rectosigmoid and uterosacral ligament involvement by endometriosis. The twisting sign also correlates with indirect markers of pelvic adhesions, such as the absence of the posterior sliding sign and medial ovarian fixation. These findings highlight the diagnostic value of uterine malposition as an indirect indicator of DE.

Retrodisplacement of the uterus, characterized by retroflexion and/or retroversion, is typically considered a physiological variant occurring in approximately 20% of women.^19^ However, it has also been linked to posterior DE and adhesions.^20^ While retroflexion has been associated with increased menstrual pain intensity,^21^ uterine rotation along the transverse axis remains underexplored. Mechanical forces exerted by DE nodules, adhesions, and fibrotic reactions likely contribute to uterine rotation, displacing it from its typical anatomical position (Figure 3).^6^

TVS is a critical tool for diagnosing posterior compartment endometriosis, with sensitivities for rectosigmoid DE ranging from 79% to 98% when performed by skilled operators.^22^ The use of standardized assessment protocols, such as those outlined by the IDEA consensus, further optimize the diagnostic accuracy of TVS for DE.^23^ Indirect indicators, or soft markers, are relatively easy to identify and can be detected even by less‐experienced sonographers for suspecting the presence of DE in patients with dysmenorrhea and chronic pelvic pain. Among them, the posterior sliding sign has been extensively validated as a reliable indirect marker of DE, with studies reporting sensitivities of up to 88% and specificities of approximately 94% for detecting obliteration of the POD, along with similarly high accuracy for identifying bowel involvement, especially rectosigmoid lesions.^24^ Moreover, ovarian immobility has also been shown to correlate with advanced‐stage and surgically confirmed DE, with a reported sensitivity of 71% for predicting the need for laparoscopic pelvic sidewall dissection.^25^ Rectosigmoid involvement has been reported in up to 92.6% of patients with endometriosis who present with the kissing ovaries sign on TVS.^13^

In our study, the twisting sign was identified in 24.1% of participants and was significantly associated with posterior compartment DE, particularly involving rectosigmoid and uterosacral ligament nodules. Notably, the likelihood of rectosigmoid junction involvement increased nearly 10‐fold in the presence of the twisting sign. This observation supports the hypothesis that mechanical traction exerted by posterior DE nodules may underlie the uterine axis rotation detected on ultrasound. As such, the twisting sign may serve as a valuable static complementary soft marker, enriching the current sonographic toolkit for raising preoperative suspicion of DE, particularly when established dynamic markers yield equivocal findings. The observed correlation between uterine rotation and the absence of the posterior sliding sign further suggests that uterine malposition reflects the extent of posterior adhesions, consistent with prior evidence linking the sliding sign to both DE and POD obliteration.^26^ Nevertheless, the lack of association between the direction of uterine rotation and the laterality of DE nodules implies that this finding is more indicative of the overall burden of posterior disease rather than the specific site of involvement.

The presence of diffuse adenomyosis with moderate to severe extension, observed more frequently in the twisting sign group (24.1% vs. 7.8%; Figure S2), further supports an association between uterine malposition and underlying pelvic pathology. By altering uterine morphology and reducing mobility, adenomyosis may contribute to displacement of the uterine axis. Its well‐established association with endometriosis may also amplify pelvic anatomical distortion. While these findings are consistent with the MUSA criteria for diagnosing adenomyosis,^10^ we consider these signs to be nonspecific, as the criteria grade severity based on the extent of myometrial involvement rather than the number or type of features. This variability in uterine morphology among reproductive‐aged women may lead to misdiagnosis and limits the generalizability and interpretability of our data in this subgroup.

Unlike three‐dimensional sonography, which has been previously used to attempt measurements of uterine rotation relative to the horizontal plane,^7^ our study employed a simplified and reproducible method to identify the twisting sign during routine TVS, without requiring complex calculations. Additional strengths include the prospective enrollment of participants and the use of standardized assessments based on the IDEA consensus,^9^, ^10^ conducted by a single experienced operator—factors that enhance methodological rigor and internal validity. However, these same conditions may also limit the generalizability of our findings. As with other sonographic soft markers of endometriosis, recognition of the twisting sign may be influenced by operator expertise, and its identification in routine or nonspecialized settings may carry a risk of both underdetection and overdetection. Broader validation among operators and institutions is needed to assess its reproducibility and diagnostic performance in settings with varying levels of experience in TVS for DE.

First, the threshold of 15° used to define the twisting sign was based on preliminary clinical observations and may not fully capture the pathological spectrum. Although a strong association with the posterior compartment DE was observed, causality cannot be inferred. Moreover, this study was not designed to include systematic surgical confirmation, as its primary aim was to characterize a novel ultrasonographic marker in a nonsurgical population. In a small subgroup of patients who underwent surgery for independent clinical indications, the twisting sign was frequently accompanied by intraoperative findings of posterior DE. While these exploratory data are not generalizable, they may inform future hypothesis‐driven validation. In addition, the specificity of the twisting sign for DE also remains to be determined, as similar uterine malpositions may occur in other pelvic conditions, such as pelvic inflammatory disease or fibrotic distortion. Although no a priori sample size calculation was performed, a post hoc power analysis supported the adequacy of the sample for exploratory purposes. Finally, as clinical practice increasingly restricts laparoscopy to selected indications, in line with current European Society of Human Reproduction and Embryology (ESHRE) guidelines,^27^ future studies must balance the need for histological confirmation with evolving standards of care. Multicenter validation studies will be essential to confirm and refine the diagnostic utility of this potential sonographic marker.

## AUTHOR CONTRIBUTIONS

Conceptualization: FB and UP. Formal analysis: GE, UP, and ULRM. Investigation: FB and UP. Methodology: SF, AP, and AI. Supervision: SF, ULRM, and SB. Writing—original draft: FB, UP, and SF. Writing—review & editing: AI and SB.

## CONFLICT OF INTEREST STATEMENT

The authors declare that they have no conflicts of interest.

## Supporting information


**Figure S1.** Flowchart of the study.

**Figure S2.** Ultrasonographic appearance of the “twisting sign” with a significant uterine axis rotation of 37.5° to the right. The image highlights diffuse uterine adenomyosis, characterized by small myometrial cysts and hyperechogenic islands. A 13 × 8‐mm deep endometriotic nodule is visible in the right uterosacral ligament (USL), extending into the ipsilateral posterior parametrium (PP). The right ovary (O) is fixed to the uterine body. In addition, a 14 × 4‐mm endometriotic plaque (°) is identified at the rectosigmoid junction, with initial muscular bowel infiltration (arrow).

Table S1.

File S1.

Video S1.

Video S2.

## Data Availability

Data is available from the authors upon reasonable request.
