# Supplementary figures and images for: Uterine “twisting sign”: A new potential ultrasonographic soft marker for deep endometriosis
Source: Int J Gynaecol Obstet. 2025 Jun 14;171(3):1355–63. doi: 10.1002/ijgo.70274 (PMC12640171; doi:10.1002/ijgo.70274)

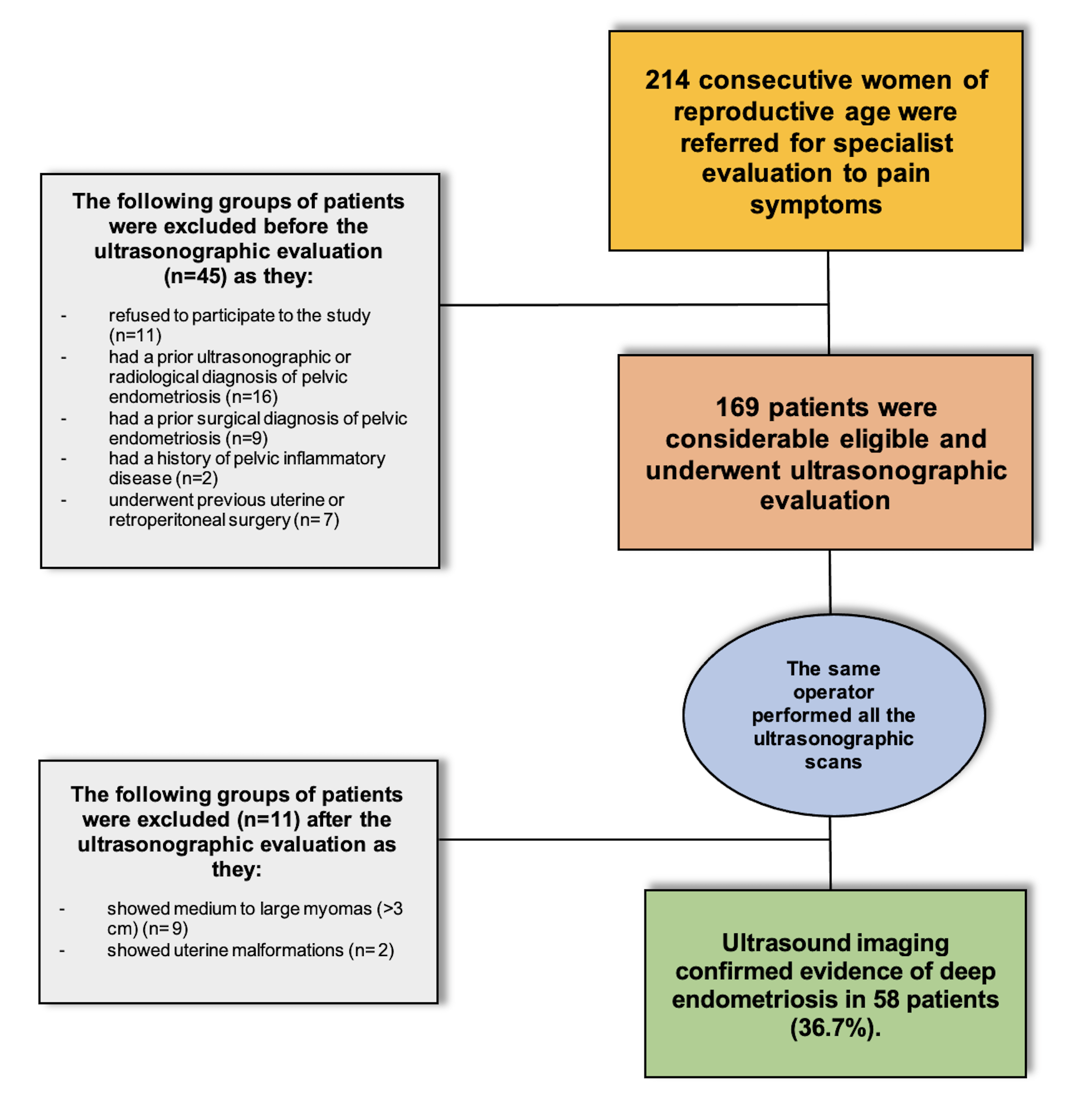

Supplement: Supplementary file 1 — Figure S1. Flowchart of the study. [file IJGO-171-1355-s001.jpg]

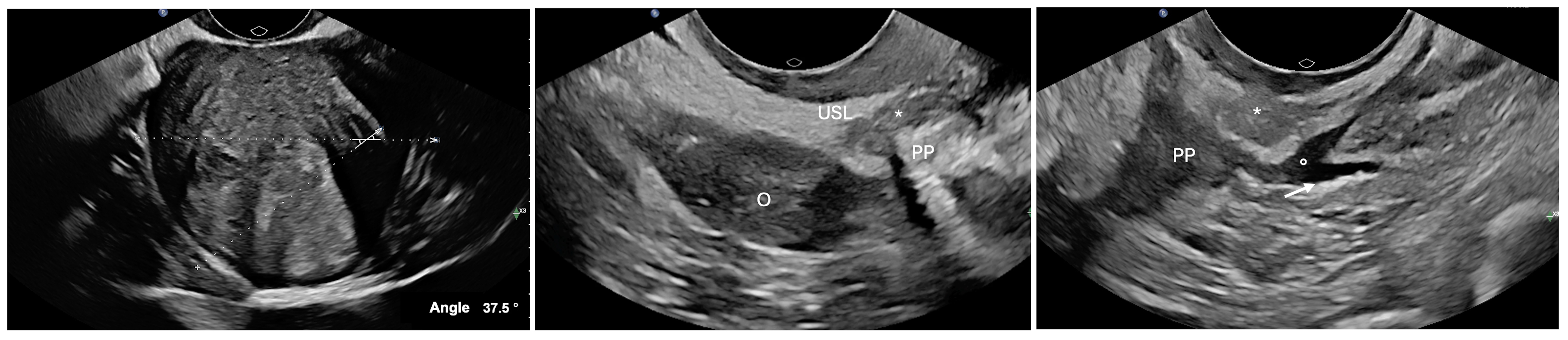

Supplement: Supplementary file 2 — Figure S2. Ultrasonographic appearance of the “twisting sign” with a significant uterine axis rotation of 37.5° to the right. The image highlights diffuse uterine adenomyosis, characterized by small myometrial cysts and hyperechogenic islands. A 13 × 8‐mm deep endometriotic nodule is visible in the right uterosacral ligament (USL), extending into the ipsilateral posterior parametrium (PP). The right ovary (O) is fixed to the uterine body. In addition, a 14 × 4‐mm endometriotic plaque (°) is identified at the rectosigmoid junction, with initial muscular bowel infiltration (arrow). [file IJGO-171-1355-s002.jpg]
