# Supplementary material for: Uterine “twisting sign”: A new potential ultrasonographic soft marker for deep endometriosis
Source: Int J Gynaecol Obstet. 2025 Jun 14;171(3):1355–63. doi: 10.1002/ijgo.70274 (PMC12640171; doi:10.1002/ijgo.70274)
Supplement: Supplementary file 3 — Table S1. [file IJGO-171-1355-s003.docx]

**Supplementary Table 1.**

| **PATIENTS WITH ULTRASONOGRAPHIC EVIDENCE OF:** | **EVIDENCE OF “TWISTING SIGN”** | **NO EVIDENCE OF “TWISTING SIGN”** | **TOTAL** | **P** |
| --- | --- | --- | --- | --- |
| **Anteverted uterus** (n, %) | 23 (79.3%%) | 90 (68.9%) | 113 (71.5%) | 0.304 |
| **Retroverted uterus (**n, %) | 6 (20.7%) | 33 (25.6%) | 39 (24.7%) | 0.581 |
| **Euverted uterus** (n, %) | 0 (-) | 6 (4.7%) | 6 (3.8%) | 0.236 |
| **Uterine length** (mm; mean ± SD) | 69.6 ± 8.5 | 68.9 ± 10.3 | 69.0 ± 10.0 | 0.732 |
| **Uterine width** (mm; mean ± SD) | 16.1 ± 3.4 | 17.4 ± 4.4 | 17.2 ± 4.3 | 0.127 |
| **Uterine height** (mm; mean ± SD) | 35.5 ± 4.5 | 35.4 ± 4.7 | 35.5 ± 4.7 | 0.938 |
